# 1459. Association of Antibiotic Prophylaxis Duration and Length of Stay in Patients Undergoing Coronary Artery Bypass Graft Procedure

**DOI:** 10.1093/ofid/ofad500.1296

**Published:** 2023-11-27

**Authors:** Shaikha Alnaimi, Warda Obaidi, Masa Habra, Bassam Shoman, Cornelia Carr, Moza Alhail

**Affiliations:** Hamad Medical Corporation, Doha, Ad Dawhah, Qatar; Hamad Medical Corporation, Doha, Ad Dawhah, Qatar; Hamad Medical Corporation, Doha, Ad Dawhah, Qatar; Hamad Medical Corporation, Doha, Ad Dawhah, Qatar; Hamad Medical Corporation, Doha, Ad Dawhah, Qatar; Hamad Medical Corporation, Doha, Ad Dawhah, Qatar

## Abstract

**Background:**

Antibiotic prophylaxis in surgery is a standard measure to reduce the risk of microbial contamination following an incision. For most surgeries, a single dose of antibiotics is generally required. However, in patients undergoing coronary artery bypass graft (CABG) procedure, antibiotics have been administered for ≥ 24 hours as data on the optimal duration is not sufficient. We aimed to correlate the duration of antibiotic prophylaxis in patients undergoing CABG with length of hospital stay (LOS).

**Methods:**

We conducted a retrospective cohort study comparing LOS of patients undergoing CABG who received antibiotic prophylaxis for ≥ 24 hours (extended course group) and those who received it for < 24 hours (short course group). Inclusion criteria were adults who had undergone CABG between 01/09/2015 –01/03/2019 in a cardiology and cardiothoracic surgery hospital. Exclusion criteria were immunocompromised patients, in-hospital mortality and patients with a pre-existing infection. The primary outcome was LOS and we also explored other variables that may predict LOS. Effects were adjusted for confounders using a multivariate linear regression model.

**Results:**

A total of 855 patients were identified. After exclusion, 840 were included in the analysis of which 425 (50.6%) received antibiotics for ≥ 24 hours and 415 (49.4%) received them for < 24 hours. The median LOS was 12.2 [IQR 11.0] days in the extended course group and 8.4 [IQR 8.8] days in the short course group, with a difference of 3.8 days (95% CI: 1.8-5.7), p-value < 0.001. However, after adjusting for potential confounders the difference was no longer significant, 1.7 days (95% CI: -0.23-3.7), p-value 0.083. Independent predictors of length of stay were, male gender, presence of chronic kidney disease, American Society of Anesthesiologists class, priority of the surgery and history of infection in the last 90 days.

**Conclusion:**

There is no statistically significant difference in LOS between short and extended antibiotic prophylaxis course in patients undergoing CABG procedure. Future work should explore the cost-effectiveness of short versus extended course antibiotic prophylaxis.

**Disclosures:**

**All Authors**: No reported disclosures

